# Neuromuscular Scoliosis as a Rare Manifestation of Guillain-Barré Syndrome in a Child

**DOI:** 10.7759/cureus.100674

**Published:** 2026-01-03

**Authors:** Spyridon I Antonopoulos, Anastasia Pilichou, Eleni Pappa, Panagiotis Krallis

**Affiliations:** 1 2nd Department of Pediatric Orthopeadics, Agia Sofia Children's Hospital, Athens, GRC; 2 2nd Department of Pediatric Orthopaedics, Agia Sofia Children's Hospital, Athens, GRC

**Keywords:** case report, flaccid paralysis, fusionless spinal surgery, guillain-barré syndrome, iliosacral fixation, minimally invasive bipolar fixation, neuromuscular scoliosis, pediatric scoliosis

## Abstract

Guillain-Barré syndrome (GBS) is an acute autoimmune polyneuropathy that may lead to long-term neuromuscular deficits, though the development of scoliosis as a late complication is rare, especially in children. We present the case of a nine-year-old boy with progressive thoracic scoliosis following a severe episode of GBS diagnosed at 18 months of age, which resulted in persistent flaccid paralysis, bilateral hip dislocation, and long-term tracheostomy-assisted respiratory support. Radiographs revealed a 71° thoracic curve, and the patient underwent correction using a minimally invasive bipolar fixation (MIBF) fusionless technique that incorporated iliosacral connectors, lumbar pedicle screws, and thoracic hooks, enabling future lengthening. Postoperative imaging showed a reduction of the curve to 32°, achieving a 54.93% correction, and the patient experienced an uncomplicated recovery with stable alignment at one-year follow-up. This case highlights the potential for neuromuscular scoliosis to develop after childhood GBS and supports the use of fusionless minimally invasive techniques in managing spinal deformity in young, medically complex patients.

## Introduction

Guillain-Barré syndrome (GBS) is an acute autoimmune inflammatory demyelinating polyneuropathy that primarily affects the peripheral nervous system and represents the most common cause of acute flaccid paralysis worldwide [1]. Its incidence is estimated at 1.2 to 3 cases per 100,000 individuals annually, with approximately 100,000 new cases reported globally each year [2,3]. Several factors have been implicated in the pathogenesis of this condition, including immunization, infections, trauma, and surgical procedures [4]. Among infectious triggers, *Campylobacter jejuni* is the most frequently reported causal pathogen in the literature [5]. Diagnosis is mainly clinical, supported by cerebrospinal fluid analysis and nerve conduction studies. GBS is a serious disorder requiring close monitoring due to its potential for rapid progression and life-threatening complications. Treatment is primarily supportive, with intravenous immunoglobulin and plasma exchange being the only therapies shown to accelerate recovery [6].

Surgery is a well-recognized precipitating factor for GBS and has been widely documented in the literature [7]. Acute postoperative onset has been reported following multiple spinal surgeries, elective spine procedures, cervical spine operations, and in patients undergoing spine surgery for incomplete cervical cord injury with respiratory failure [8-12]. Although an association between GBS and scoliosis has been described, it remains uncommon [13]. After an extensive review of the literature, we identified only a small number of reported cases of neuromuscular scoliosis developing after GBS [14-16].

In this article, we report the case of a nine-year-old child who developed progressive neuromuscular scoliosis following a diagnosis of GBS in early childhood.

## Case presentation

We present the case of a nine-year-old male patient, admitted to Agia Sophia General Children’s Hospital in Athens for evaluation and management of progressive scoliosis. His medical history was significant for acute flaccid paralysis of both lower limbs and respiratory failure at 18 months of age, for which he was diagnosed with GBS, following prompt clinical and laboratory assessment.

Unfortunately, his neurological condition remained unchanged over time. Three months after diagnosis, he developed an acute respiratory infection requiring tracheostomy placement, followed by a long-term mechanical respiratory support. During the time of assessment, respiratory support was used only during nighttime.

**Figure 1:**
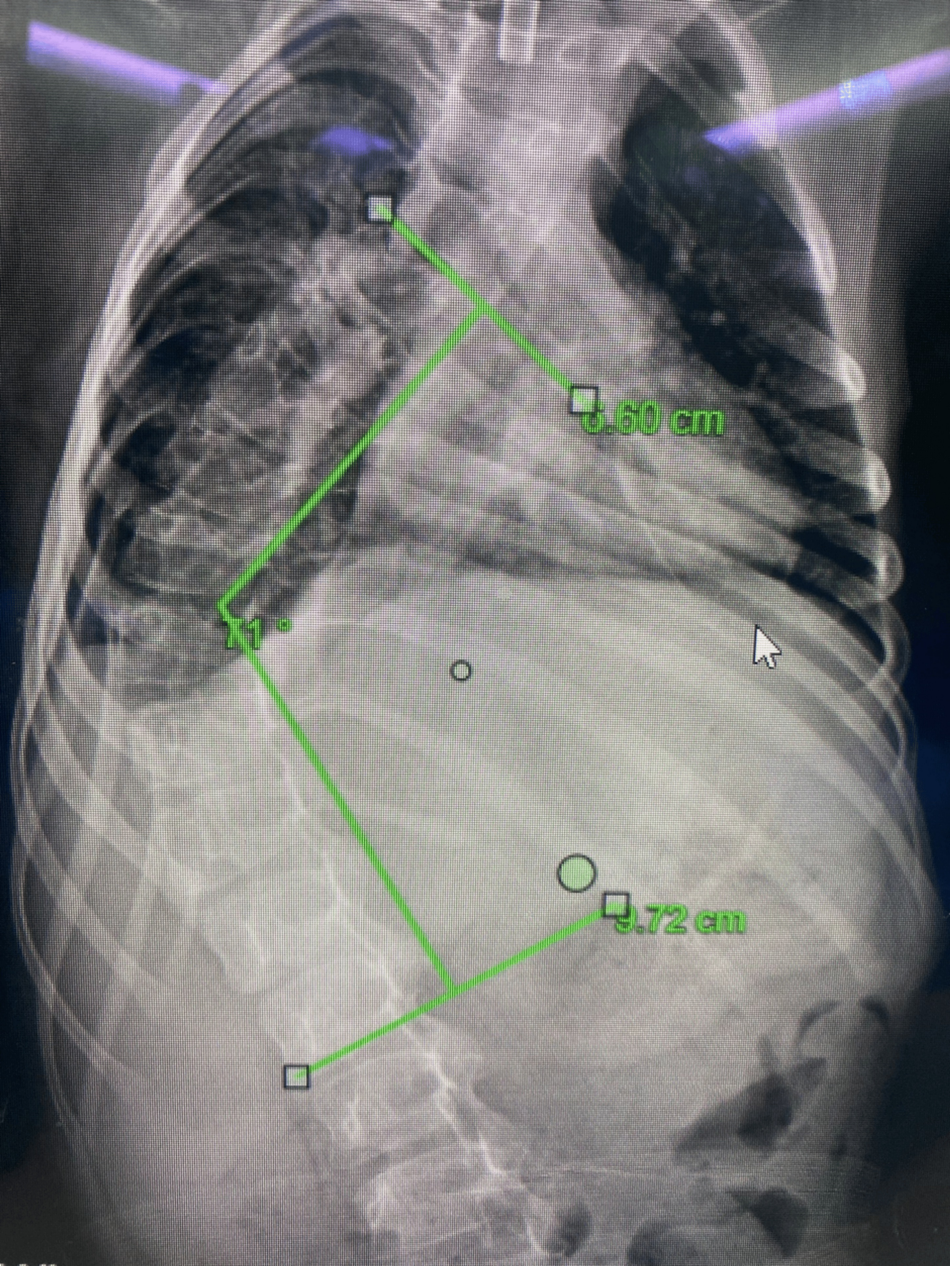
Preoperative AP thoracic and lumbar spine radiograph depicting the preoperative Cobb angle AP: Anteroposterior

**Figure 2:**
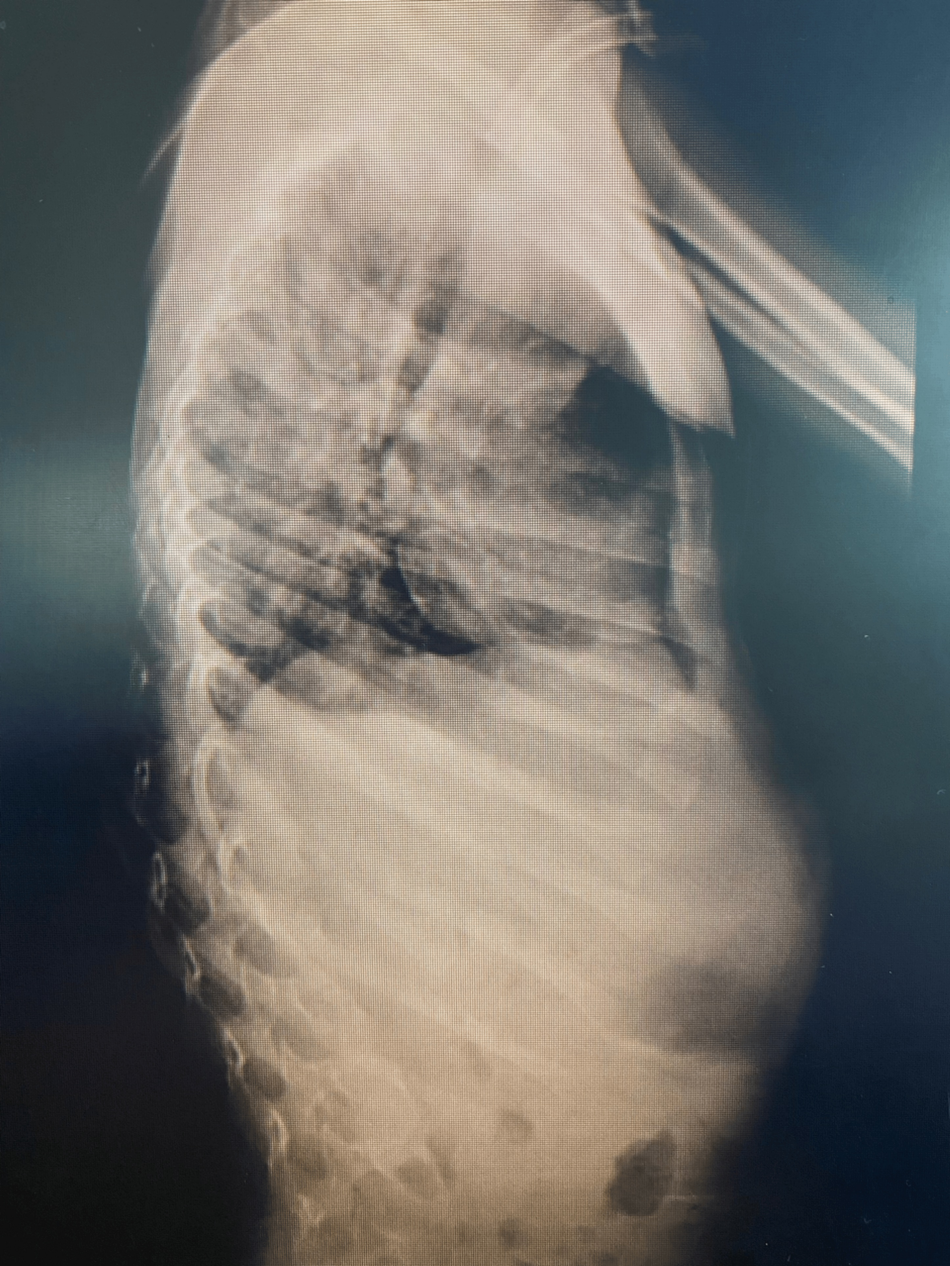
Preoperative lateral view of spine radiograph

**Figure 3:**
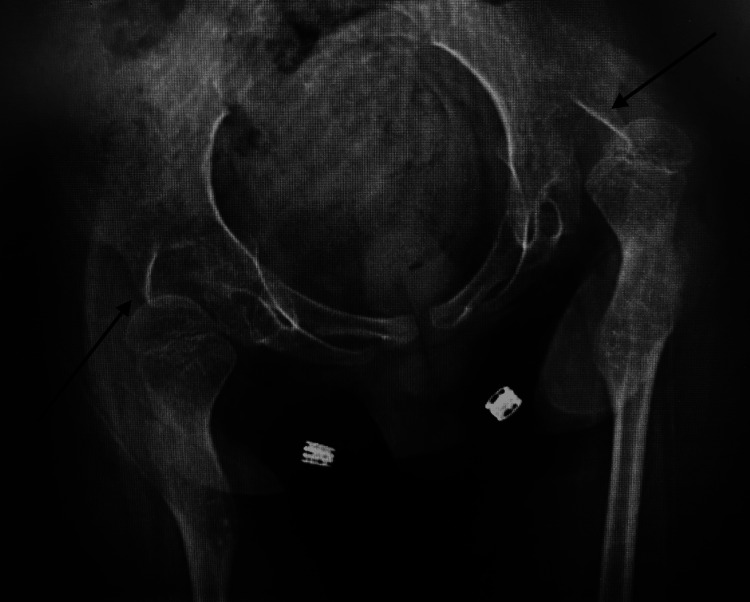
AP view pelvis radiograph with bilateral hip dislocation present as shown by the arrows AP: Anteroposterior

The patient had been under regular pediatric follow-up. Thus, three months before presentation to our clinic, he was referred due to noticeable progression of a spinal deformity. Neurologically, no improvement had occurred since the initial GBS diagnosis, while he was still non-ambulatory. Imaging evaluation included plain radiographs which revealed a 71° thoracic Cobb angle (Figures 1, 2), indicating the need for surgical correction. Bilateral hip dislocation was also present (Figure 3).

Detailed preoperative planning occurred, together with an extended preoperative evaluation of the patient. Eventually, the patient underwent posterior spinal correction using a non-fusion minimally invasive bipolar fixation (MIBF) technique. More specifically, two short posterior incisions were used with the patient in prone position under general anesthesia, applying lower-limb traction and full intra-operative neuromonitoring. Given his non-ambulatory status, the pelvis was included in the construct. A short midline incision at the lumbosacral junction allowed exposure of the sacrum through a Wiltse approach. In that way, bilateral sacral cortical entry points were created lateral to the S1 articular process and above the first posterior sacral foramen. Then iliosacral connectors were inserted, followed by percutaneous iliosacral screw placement in an oblique anteroposterior trajectory to avoid the spinal canal.

**Figure 4:**
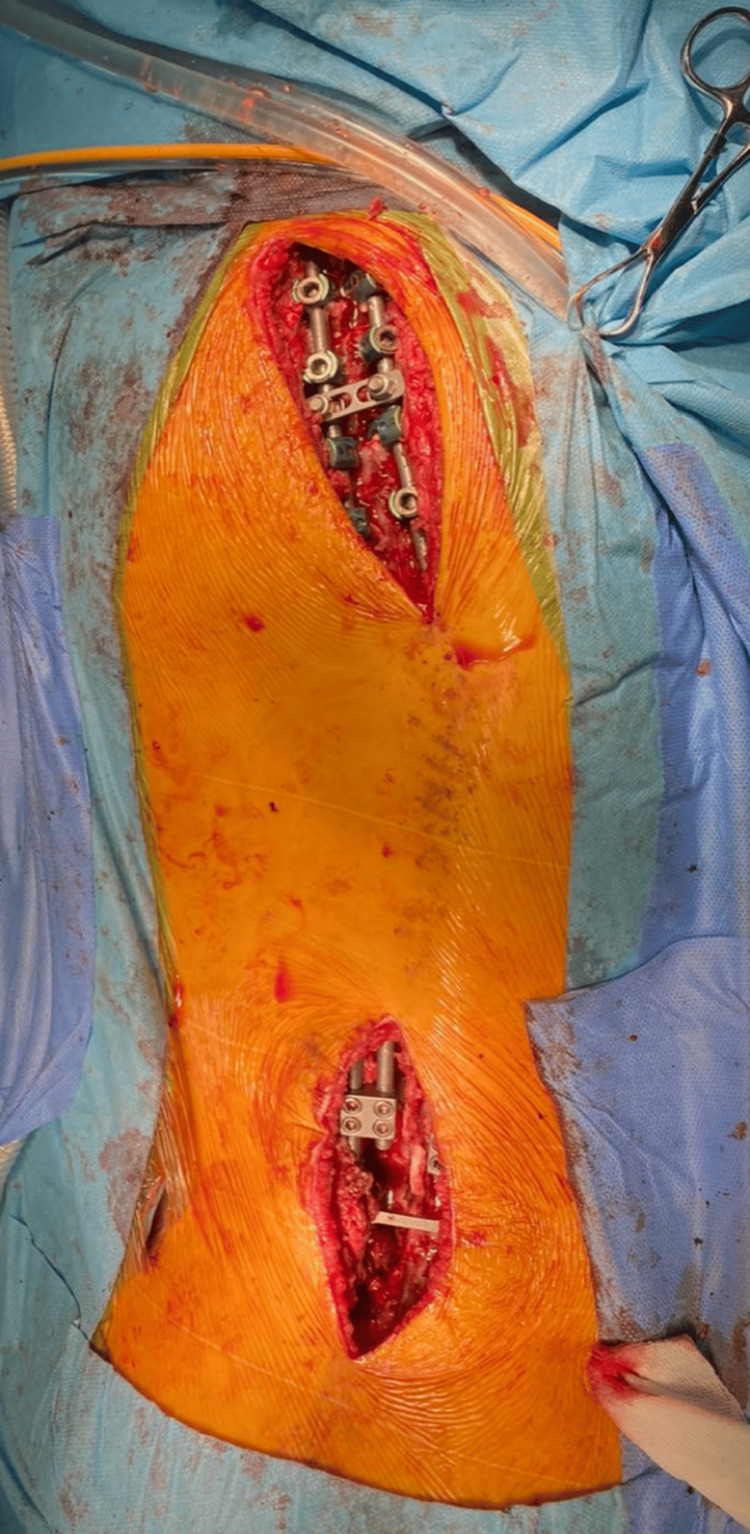
Intra-operative image of the surgical field with patient in prone position showing the minimally invasive non-fusion bipolar surgical technique

Furthermore, thoracic fixation was achieved through a second short midline incision. Then hooks were placed as they are better in terms of strong fixation, particularly in patients with neuromuscular disease or poor bone quality. Moreover, precontoured rods were placed in the thoracic hooks and then connected to the lumbosacral/iliosacral construct using dominos, allowing the potential for future lengthening given the patient’s age and the non-fusion nature of the technique (Figure 4).

**Figure 5:**
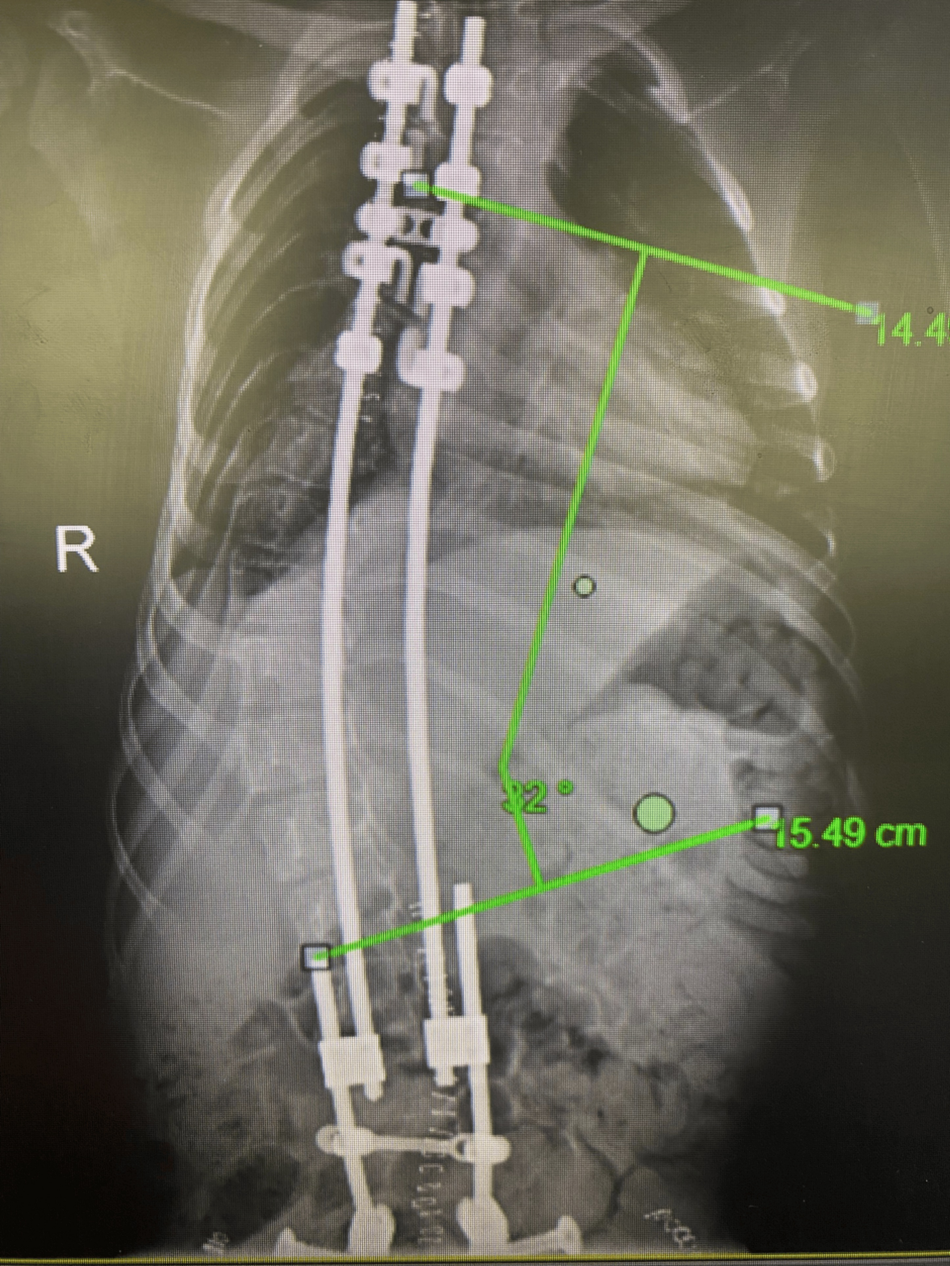
Postoperative correction of the Cobb angle on AP full-spine radiograph AP: Anteroposterior

**Figure 6:**
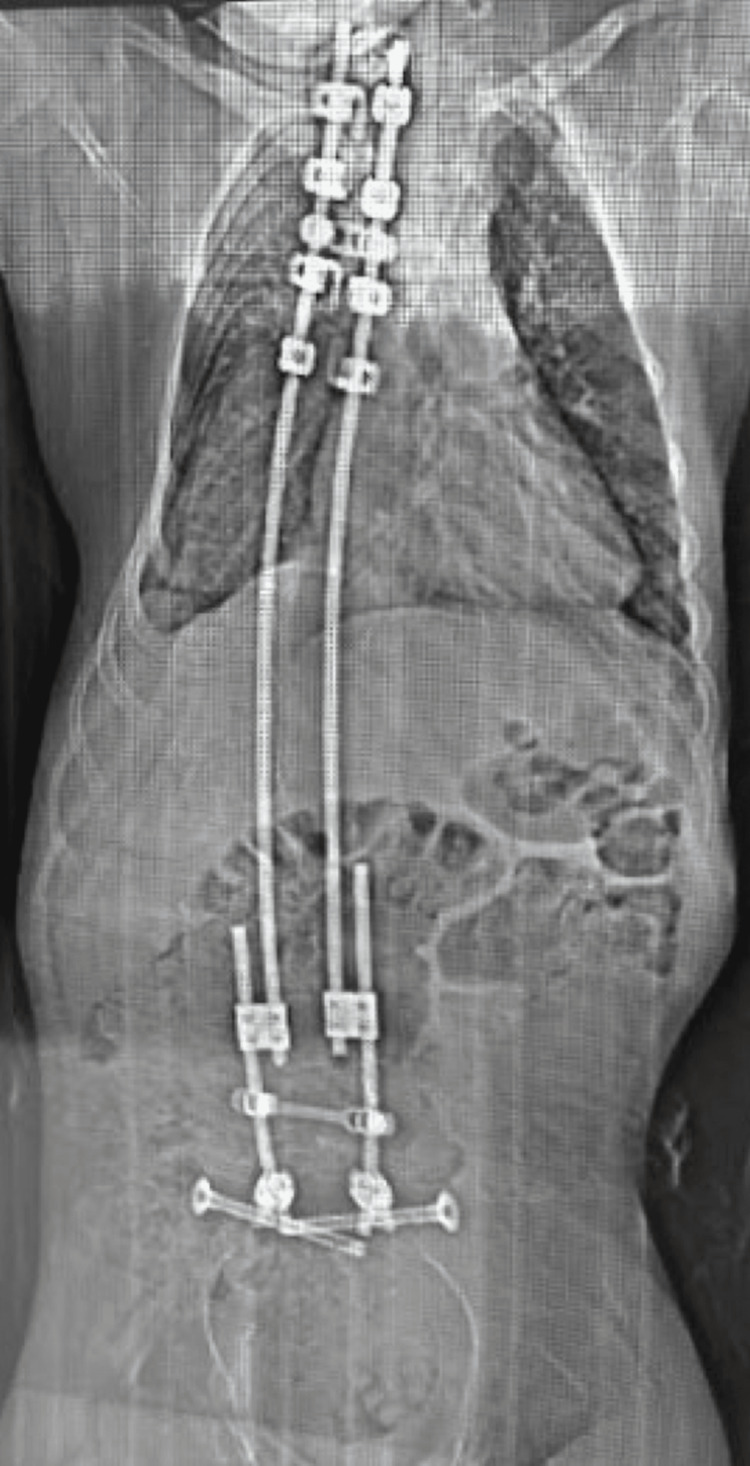
Latest follow-up AP full-spine radiograph depicting the final postoperative construct AP: Anteroposterior

**Figure 7:**
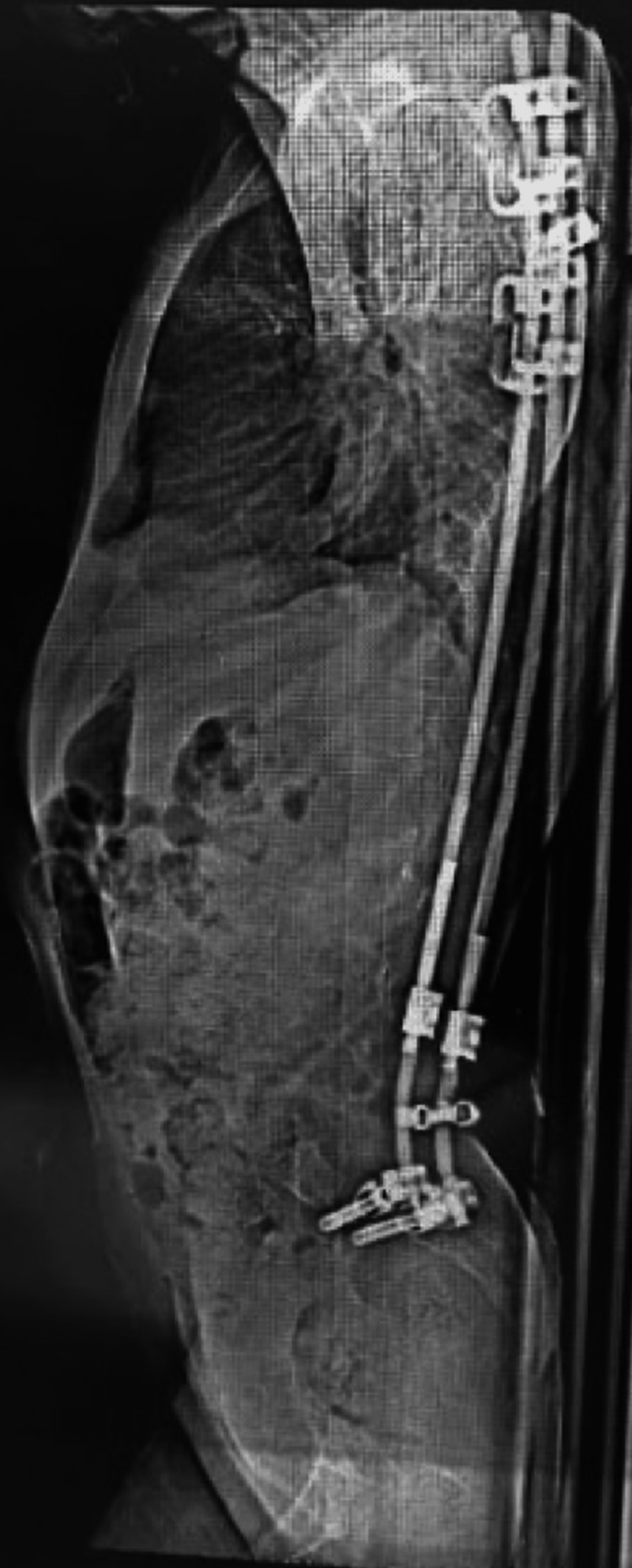
Latest follow-up lateral spine radiograph showing the proper positioning of the whole construct

Regarding the postoperative recovery, the patient signed an uneventful progress and was discharged seven days postoperatively. Postoperative imaging demonstrated satisfactory correction, reducing the thoracic Cobb angle to 32°, corresponding to a 54.93% correction rate (Figure 5). During the latest follow-up, one-and-a-half years postoperatively, the patient continues to do well and remains actively engaged in rehabilitation (Figures 6, 7).

## Discussion

GBS is an acute autoimmune polyneuropathy that typically presents with ascending weakness, often involving the lower limbs, especially in early stages [17]. While GBS is more commonly diagnosed in adults, pediatric cases do occur, though long-term musculoskeletal sequelae such as neuromuscular scoliosis are rarely reported. After reviewing the international literature, we identified only a few cases describing scoliosis secondary to GBS, underscoring the rarity of this association.

Li et al. reported successful management of a 14-year-old male patient with scoliosis secondary to GBS. The patient first underwent resection of a T4 cavernous hemangioma, followed by posterior spinal fusion from T5 to L5 three months later, achieving a 60.5% correction rate [14]. Edwards et al. described an eight-year-old child who underwent posterior correction and fusion from T2 to L1 using sublaminar hooks, pedicle screws, and rods [15].

The technique used in our case is the so called MIBF, and was introduced as a non-fusion strategy in 2010 and has since become an effective method for managing neuromuscular and early-onset spinal deformities [18]. The principle involves creating a strong, stable construct that provides satisfactory deformity correction without immediate fusion, allowing continued spinal growth and the possibility of future lengthening procedures. Long-term fusion typically occurs gradually due to prolonged rigidity of the construct. Additionally, the aforementioned technique preserves flexibility in the intervening spinal segments, delaying the need for definitive fusion which is of crucial importance for these young patients.

For rigid or severe deformities, preoperative halo-gravity traction or casting is often recommended to improve correction potential [19]. However, in this case, the patient’s overall medical condition made such preparatory interventions unsuitable.

Given the patient’s young age, non-ambulatory status, and neuromuscular etiology, MIBF offered a safe, effective, and growth-preserving solution with low morbidity, as it was shown by the patients uneventful postoperative progress.

## Conclusions

Neuromuscular complications following GBS can occur, though the development of scoliosis remains rare. We present the case of a nine-year-old child who developed progressive thoracic scoliosis following a severe episode of GBS at 18 months of age. With no established surgical guidelines for scoliosis management in this patient population, we elected to perform a fusionless MIBF procedure to achieve deformity correction while preserving spinal growth potential. Although GBS is uncommon in children, spine surgeons should remain vigilant for progressive spinal deformities in pediatric patients with significant residual neuromuscular deficits after GBS and ensure thorough musculoskeletal evaluation during follow-up.
